# Supplementary material for: Structural basis for membrane attack complex inhibition by CD59
Source: Nat Commun. 2023 Feb 16;14:890. doi: 10.1038/s41467-023-36441-z (PMC9935631; doi:10.1038/s41467-023-36441-z)
Supplement: Supplementary file 1 — Supplementary Information [file 41467_2023_36441_MOESM1_ESM.pdf]

Structural basis for membrane attack complex inhibition by CD59

Emma C. Couves[1], Scott Gardner[1], Tomas B. Voisin[1], Jasmine K. Bickel[1,2], Phillip J. Stansfeld[3], Edward W. Tate[2], Doryen Bubeck[1*]

[1] Department of Life Sciences, Sir Ernst Chain Building, Imperial College London, London SW7 2AZ, United Kingdom

[2] Department of Chemistry, Molecular Sciences Research Hub, Imperial College London, London, W12 0BZ, United Kingdom

[3] School of Life Sciences and Department of Chemistry, Gibbet Hill Campus, The University of Warwick, Coventry, CV4 7AL

*Correspondence to: d.bubeck@imperial.ac.uk

Supplementary information

Supplementary Figs. 1-9

Supplementary Table 1

Supplementary Table 2

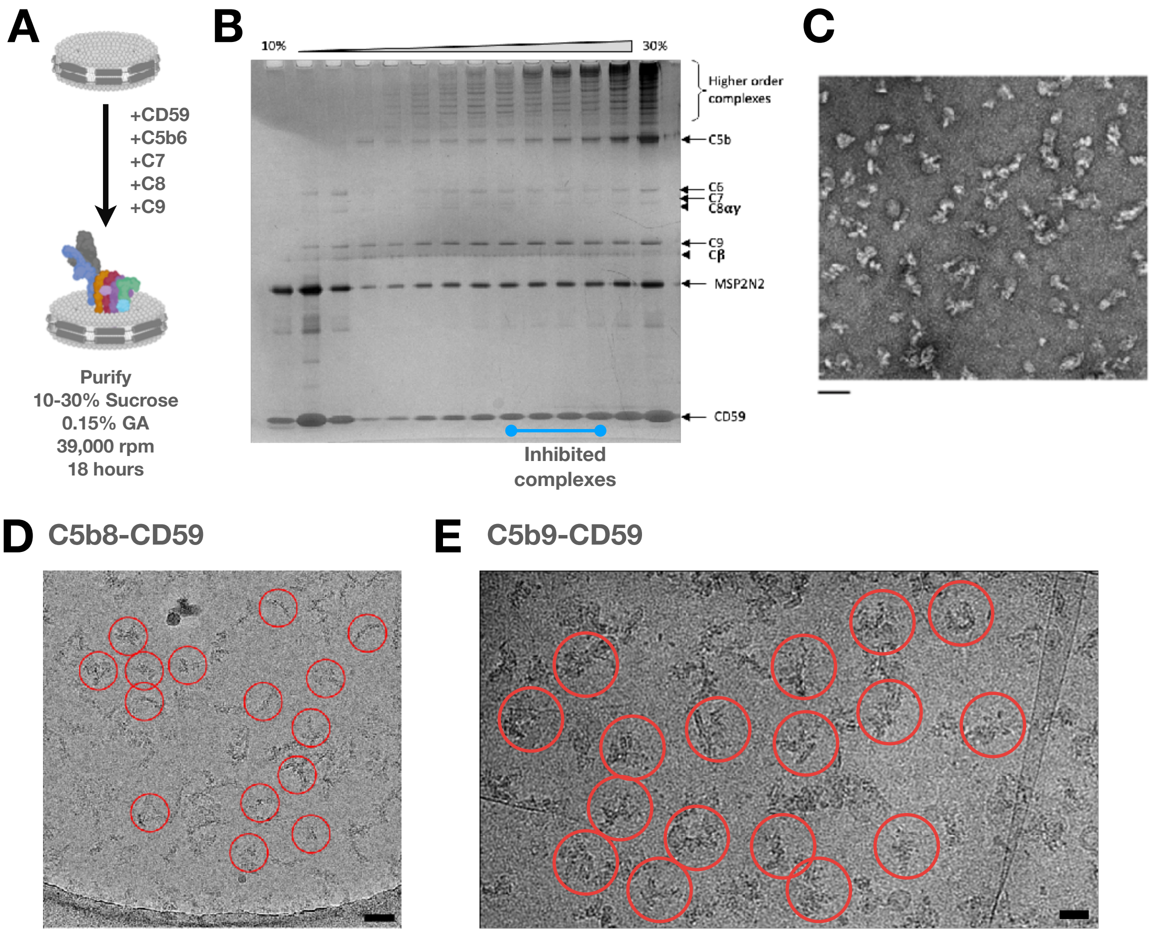

Supplementary Figure 1. Supplementary cryoEM data. (**A**) Schematic showing the assembly and purification of CD59-inhibited complement complexes on lipid nanodiscs. Image was created using Biorender (Agreement no: KD24VPIN0O) (**B**) Non-reduced SDS-PAGE gel of fractions along the density centrifugation gradient (10-30% sucrose). Sizes corresponding to individual complement proteins, CD59 and MSP2N2 from the nanodisc are indicated. Fractions pooled for further structural analysis are indicated by a blue line. Gel representative of three independent experiments. Uncropped gel can be found in the Source Data file. (**C**) Negatively stained C5b9-CD59 complexes corresponding to pooled fractions in (B). A representative micrograph from 50 randomly imaged locations is shown. Scale bar, 50 nm. (**D**) Representative cryoEM micrograph of C5b8-CD59 sample (selected randomly from 12,805 micrograph movies), individual complexes circled. Scale bar, 40 nm. (**E**) Representative

cryoEM micrograph of C5b9-CD59 sample (selected randomly from 52,838 micrograph movies), individual complexes circled. Scale bar, 20 nm.

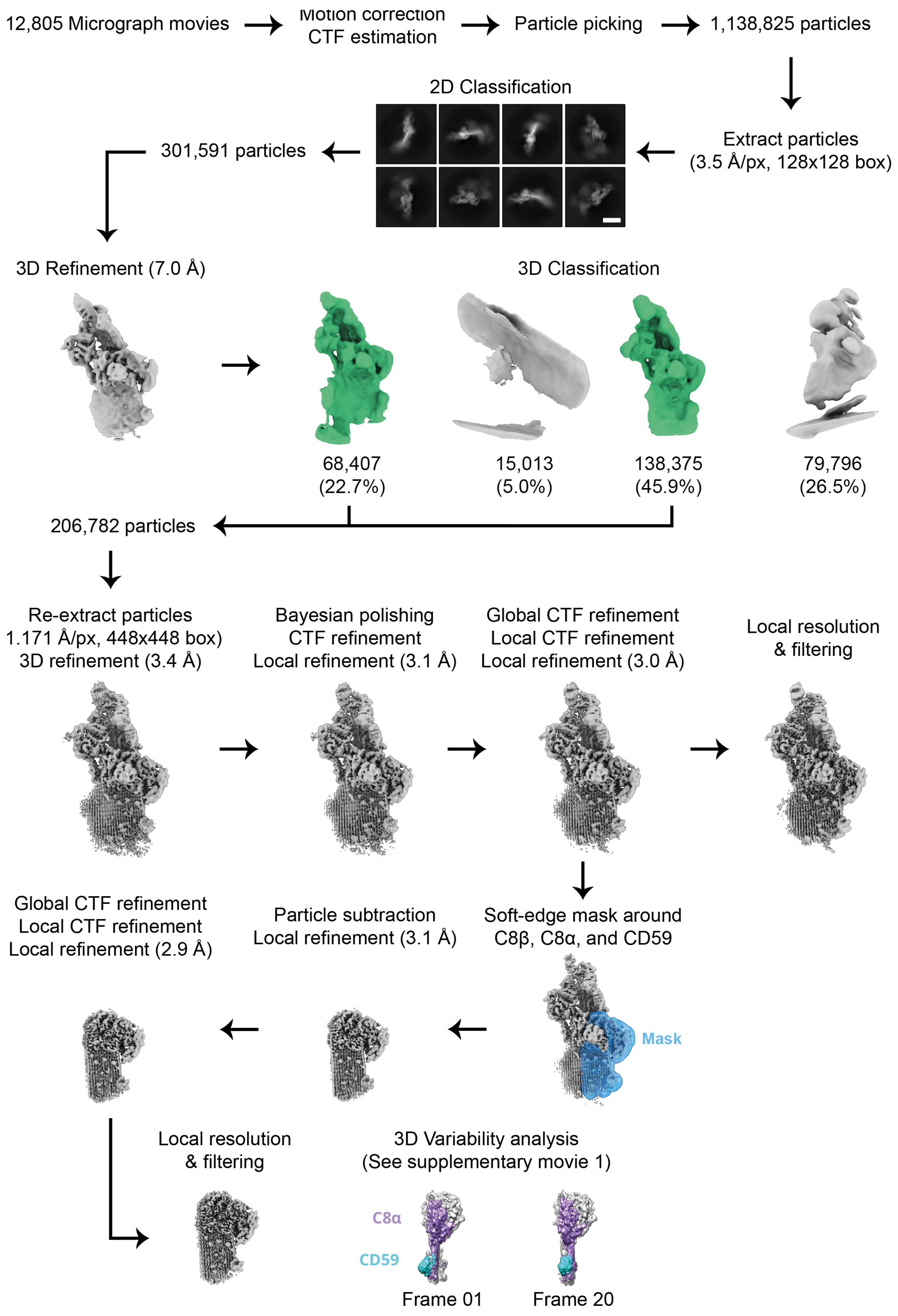

Supplementary Figure 2. CryoEM image processing workflow for C5b8-CD59 map. Schematic outlines steps performed to obtain the structure of the C5b8-CD59 complex. Scale bar referring to the 2D class averages is 13 nm. (see Methods for details).

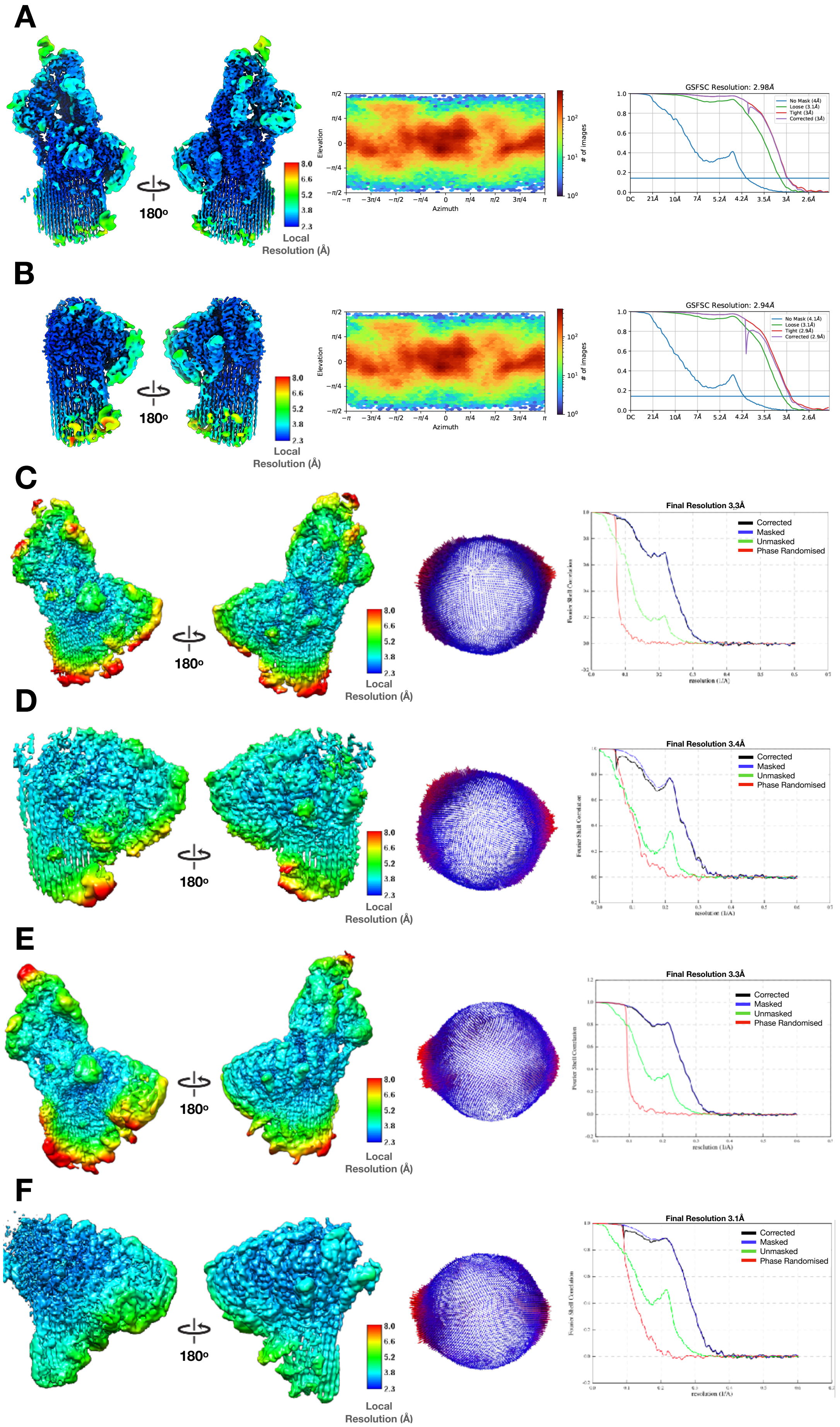

A
180°
Local Resolution (Å)
8.0
6.6
5.2
3.8
2.3
Elevation
Azimuth
# of images
GSFSC Resolution: 2.98Å
No Mask (4Å)
Loose (3.1Å)
Tight (3Å)
Corrected (3Å)
B
180°
Local Resolution (Å)
GSFSC Resolution: 2.94Å
No Mask (4.1Å)
Loose (3.1Å)
Tight (2.9Å)
Corrected (2.9Å)
C
180°
Local Resolution (Å)
Final Resolution 3.3Å
Corrected
Masked
Unmasked
Phase Randomised
D
180°
Local Resolution (Å)
Final Resolution 3.4Å
Corrected
Masked
Unmasked
Phase Randomised
E
180°
Local Resolution (Å)
Final Resolution 3.3Å
Corrected
Masked
Unmasked
Phase Randomised
F
180°
Local Resolution (Å)
Final Resolution 3.1Å
Corrected
Masked
Unmasked
Phase Randomised

Supplementary Figure 3. Map validation information for C5b8-CD59 and C5b9-CD59 complexes. Map validations for C5b8-CD59 (**A**), density subtracted focus refined C5b8-CD59 (**B**), C5b9$_2$-CD59 (**C**), density subtracted focus refined C55b9$_2$-CD59 (**D**), C5b9$_3$-CD59 (**E**), density subtracted focus refined C5b9$_3$-CD59 (**F**). Colored local resolution filtered maps for each reconstruction are shown in the left panels. Angular distribution plots for each reconstruction are shown in the middle panels. FSC curves for each reconstruction are shown in the right panels.

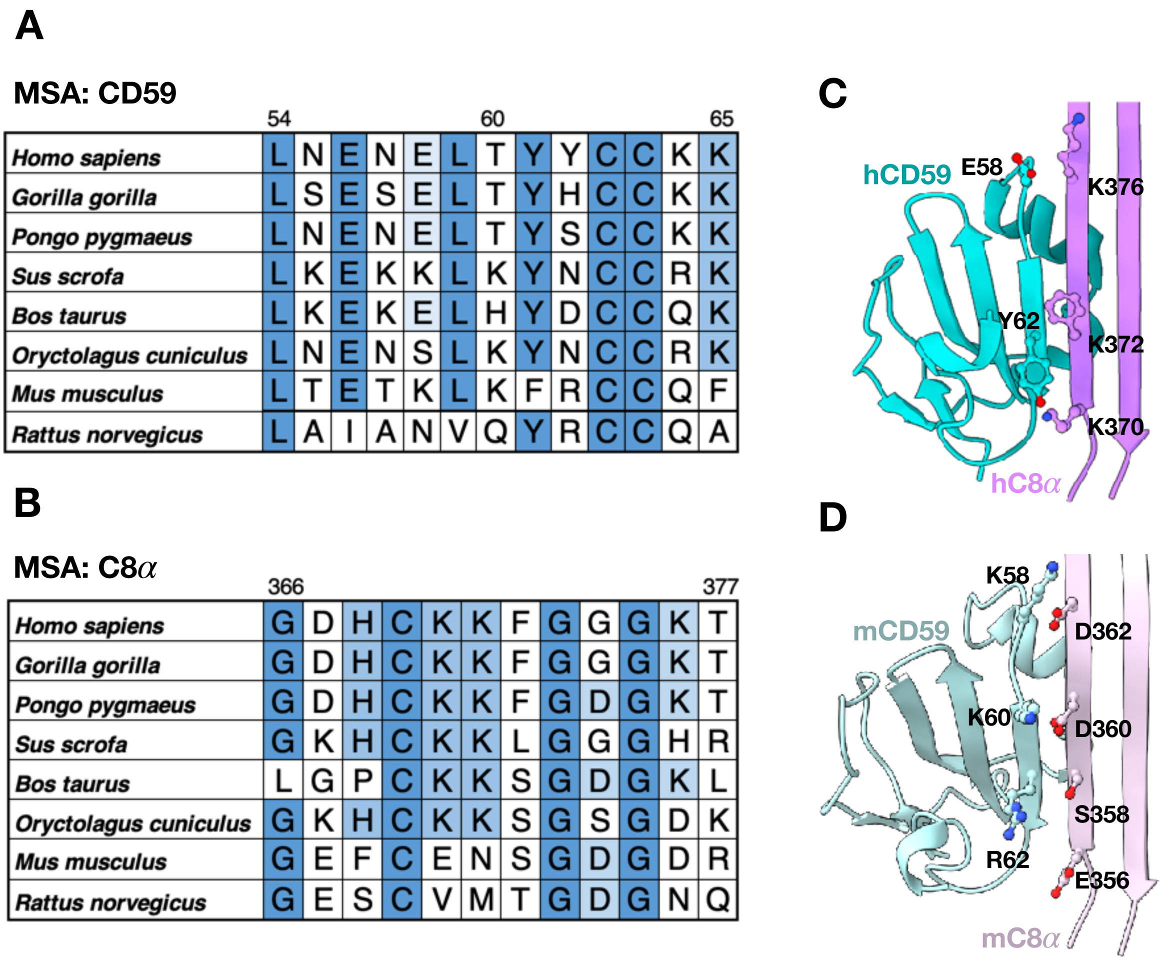

Supplementary Figure 4. Co-evolution analysis of the C8α-CD59 interface. Multisequence alignment (MSA) for CD59 (**A**) and C8α residues (**B**) within the binding interface. Numbering corresponds to the human sequence. (**C**) Ribbon diagram of the human C8α-CD59 interface in our structure. (**D**) Murine model of the interface derived from AlphaFold2 prediction of

CD59 (AlphaFold ID: O55186) and template-based model building of C8α. Key residues that mediate the interactions are shown as sticks.

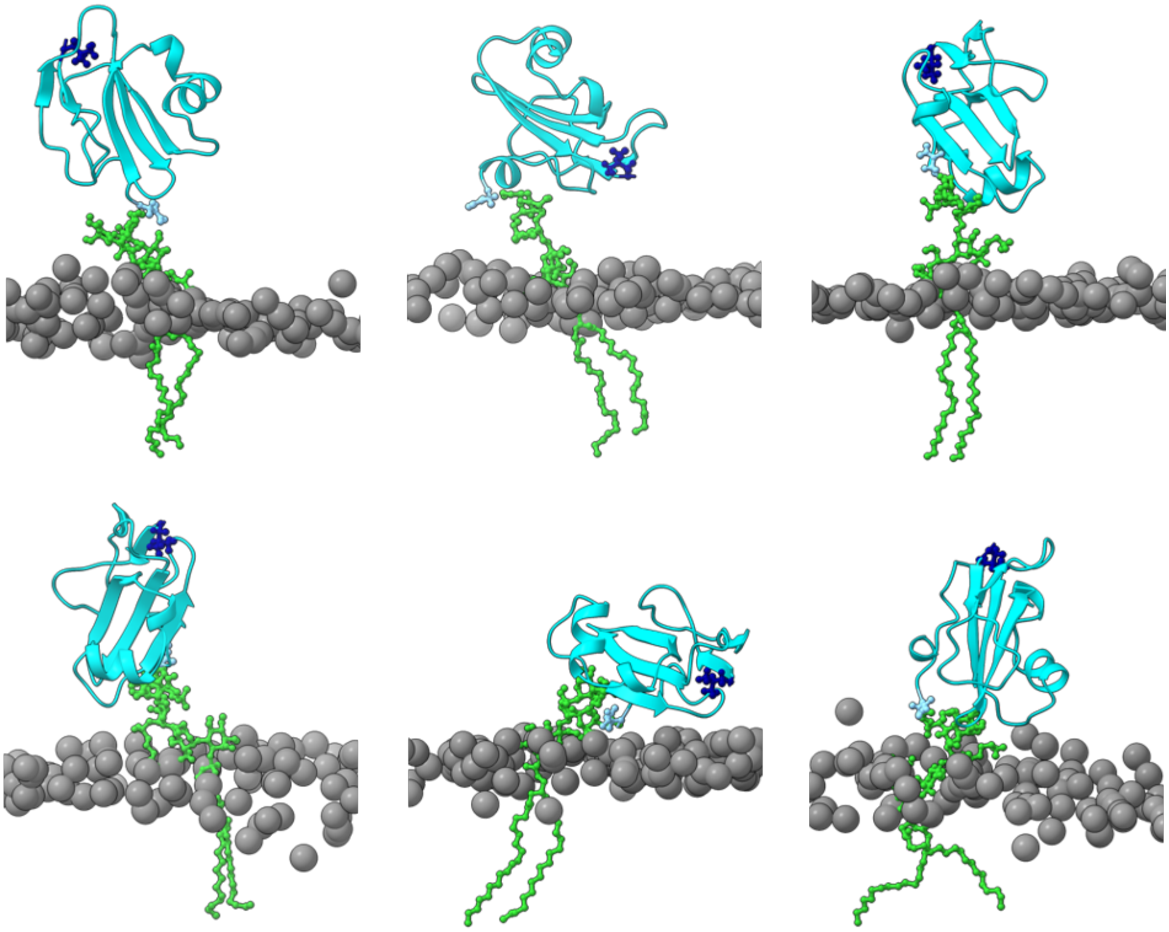

Supplementary Figure 5. MD simulations show flexibility of GPI-anchored CD59. Representative frames sampled from the atomistic MD simulations of GPI-anchored CD59 in a DOPC lipid bilayer. CD59 is shown as cyan ribbons with the GPI anchor in green sticks and the N- and C- terminal residues in dark blue and light blue sticks, respectively. Phosphorous atoms from lipid headgroups are grey spheres. Initial and final configurations for the three replicates are supplied as Supplementary Data Files 1-6.

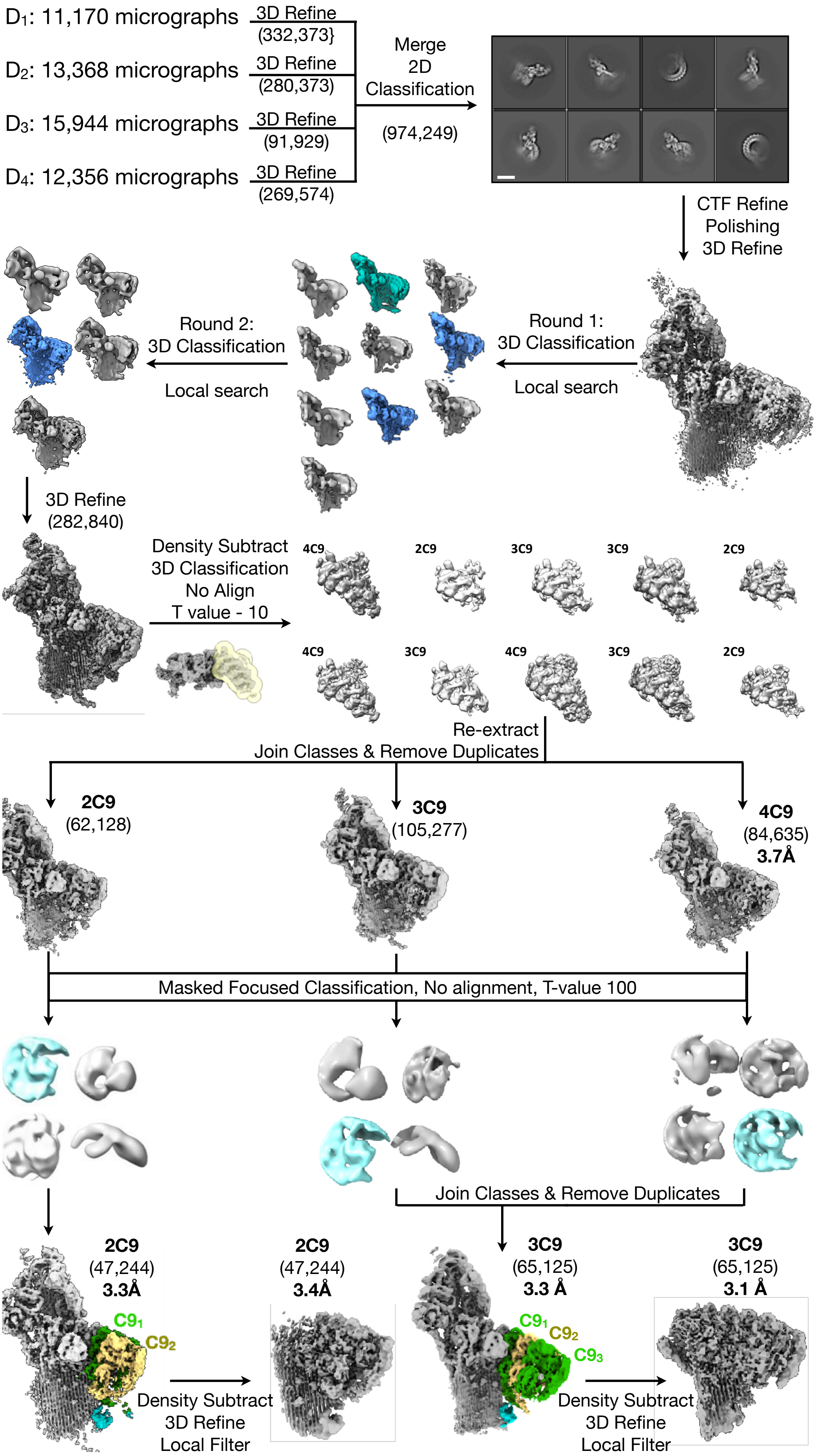

D1: 11,170 micrographs
3D Refine
(332,373}
D2: 13,368 micrographs
3D Refine
(280,373)
D3: 15,944 micrographs
3D Refine
(91,929)
D4: 12,356 micrographs
3D Refine
(269,574)
Merge
2D
Classification
(974,249)
CTF Refine
Polishing
3D Refine
Round 1:
3D Classification
Local search
Round 2:
3D Classification
Local search
3D Refine
(282,840)
Density Subtract
3D Classification
No Align
T value - 10
4C9
2C9
3C9
3C9
2C9
4C9
3C9
4C9
3C9
2C9
Re-extract
Join Classes & Remove Duplicates
2C9
(62,128)
3C9
(105,277)
4C9
(84,635)
3.7Å
Masked Focused Classification, No alignment, T-value 100
Join Classes & Remove Duplicates
2C9
(47,244)
3.3Å
C91
C92
Density Subtract
3D Refine
Local Filter
2C9
(47,244)
3.4Å
3C9
(65,125)
3.3 Å
C91
C92
C93
Density Subtract
3D Refine
Local Filter
3C9
(65,125)
3.1 Å

Supplementary Figure 6. CryoEM image processing workflow for C5b9-CD59. Schematic outlines steps performed to obtain the C5b9$_2$-CD59 and C5b9$_3$-CD59 structures. Scale bar referring to the 2D class averages is 20 nm. Particle numbers are in brackets. (see Methods for details).

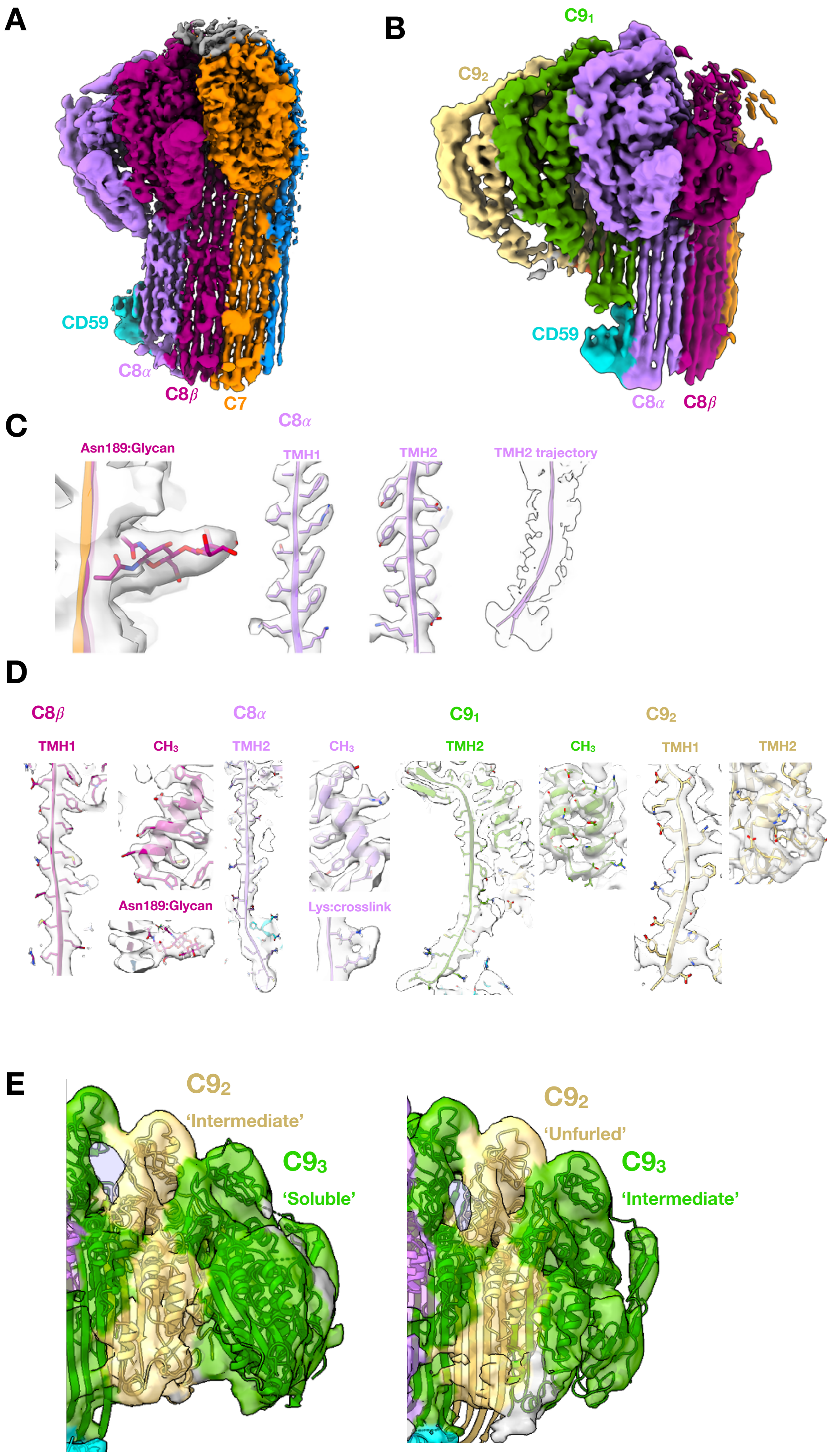
A
CD59
C8α
C8β
C7
B
C9₁
C9₂
CD59
C8α
C8β
C
C8α
Asn189:Glycan
TMH1
TMH2
TMH2 trajectory
D
C8β
TMH1
CH3
Asn189:Glycan
C8α
TMH2
CH3
Lys:crosslink
C9₁
TMH2
CH3
C9₂
TMH1
TMH2
E
C9₂
'Intermediate'
C9₃
'Soluble'
C9₂
'Unfurled'
C9₃
'Intermediate'

Supplementary Figure 7. Map quality of the C5b8-CD59 and C5b9-Cd59 structures. (**A-B**) Density subtracted focus refined maps for the C5b8-CD59 (**A**) and C5b9$_2$-CD59 (**B**) complexes. (**C-D**) Fits of the C5b8-CD59 (**C**) and C5b9$_2$-CD59 (**D**) models into their respective density subtracted maps. (**E**) Models for two conformations of the terminal C9 (C9$_3$) derived from the cyroDRGN analysis of the C5b9-CD59 complex.

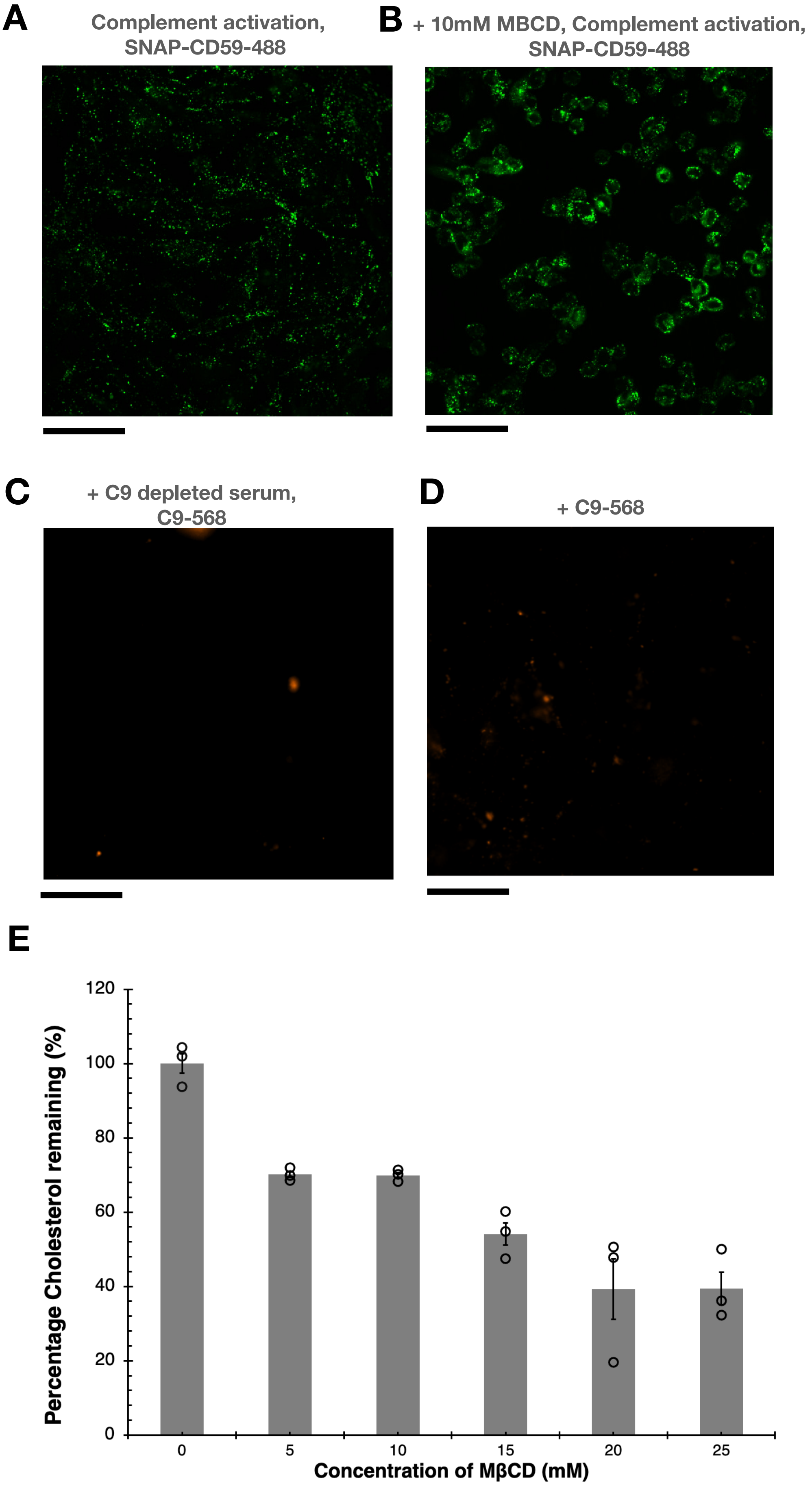
A
Complement activation,
SNAP-CD59-488
B
+ 10mM MBCD, Complement activation,
SNAP-CD59-488
C
+ C9 depleted serum,
C9-568
D
+ C9-568
E
Percentage Cholesterol remaining (%)
0
20
40
60
80
100
120
0
5
10
15
20
25
Concentration of MβCD (mM)

Supplementary Figure 8. Supporting cellular assay controls. (**A-B**) CHO cells expressing SNAP-CD59 were treated with a polyclonal anti-CHO IgG antibody to activate complement. Cells were incubated with C9-delpeted human serum supplemented with a chemically-labeled fluorescent C9 (C9-Alexafluor 568) capable of forming MAC. CD59 was visualized with SNAP-Oregon (488 nm) in control cells (**A**) and in cells treated with MβCD to deplete cholesterol (**B**). Wide-field fluorescence microscopy was used to visualize C9 on the cell surface in the absence of complement activator (**C**) and when incubated with C9-Alexafluor 568 alone (**D**). No nonspecific binding is observed. Representative images (out of 10 randomly selected locations) for condition are shown. Scale bars, 50 μm. (**E**) Amplex-Red cholesterol depletion assay detecting the extent of cholesterol present in the cells normalized to untreated cells. Individual measurements are given as points, the average and standard deviation across three technical replicates are shown. Data underlying (**E**) is found in the Source Data File.

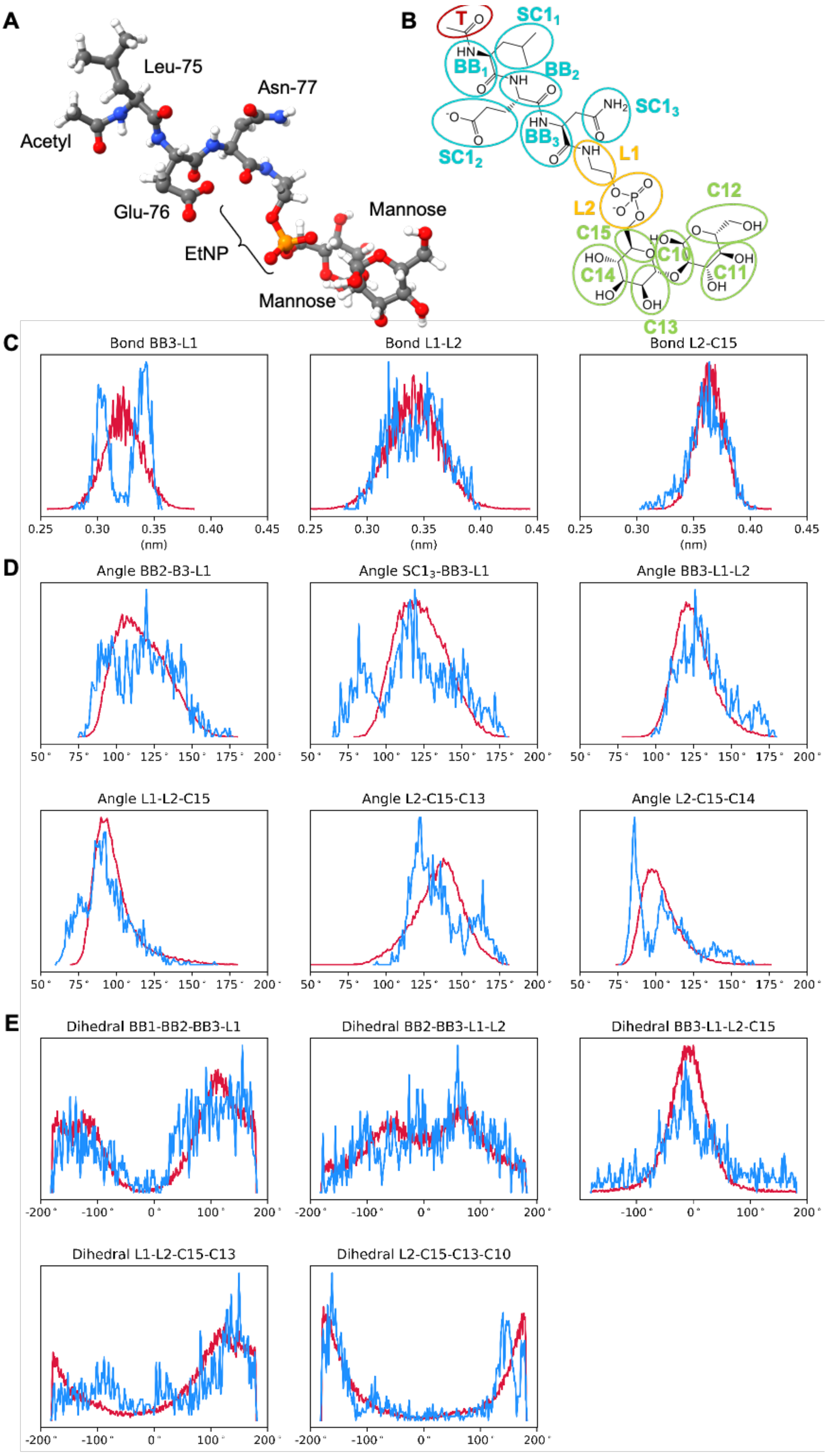

A
Leu-75
Asn-77
Acetyl
Glu-76
Mannose
EtNP
Mannose
B
C
Bond BB3-L1
Bond L1-L2
Bond L2-C15
(nm)
D
Angle BB2-B3-L1
Angle SC1₃-BB3-L1
Angle BB3-L1-L2
Angle L1-L2-C15
Angle L2-C15-C13
Angle L2-C15-C14
E
Dihedral BB1-BB2-BB3-L1
Dihedral BB2-BB3-L1-L2
Dihedral BB3-L1-L2-C15
Dihedral L1-L2-C15-C13
Dihedral L2-C15-C13-C10

Supplementary Figure 9. Parameterization of the phosphoethanolamine linker (EtNP). (**A**) Stick representation of the molecule used for parameterization. (**B**) Coarse-grained mapping of the molecule in (A). Beads corresponding to the CD59 residues are in cyan, the terminal acetyl cap is red, beads for the EtNP linker (L1 and L2) are in yellow, and beads corresponding to the mannose residues are in green. Comparing bond lengths (**C**) angles (**D**) and dihedral angles (**E**) involving beads L1 and L2 of the EtNP linker between atomistic (blue) and coarse-grained (red) simulations.

Supplementary Table 1. Validation statistics for EM maps and models.

	**#1 C5b8-CD59**<br>EMD: **15779**	**#2 C5b8-CD59^{arc}**<br>EMD: **15800**	**#3 C5b9$_2$-CD59**<br>EMD: **15781**	**#4 C5b9$_3$-CD59**<br>EMD: **15780**	**#5 C5b9$_2$-CD59^{arc}**<br>EMD: **15782**	**#6 C5b9$_3$-CD59^{arc}**<br>EMD: **15783**
**Data collection and processing**						
**Magnification**	105k	105k	105k	105k	105k	105k
**Voltage (kV)**	300	300	300	300	300	300
**Electron exposure (e–/Å^2)**	50	50	D_1 43<br>D_2 56<br>D_3 40<br>D_4 50	D_1 43<br>D_2 56<br>D_3 40<br>D_4 50	D_1 43<br>D_2 56<br>D_3 40<br>D_4 50	D_1 43<br>D_2 56<br>D_3 40<br>D_4 50
**Defocus range (μm)**	-1.0 to –2.25	-1.0 to –2.25	-1.0 to -2.25	-1.0 to -2.25	-1.0 to -2.25	-1.0 to -2.25
**Detector Type**	Falcon IV		K3	K3	K3	K3
**Pixel size (Å)**	1.171	1.171	D_1 0.829<br>D_2 0.831<br>D_3 0.831<br>D_4 0.85	D_1 0.829<br>D_2 0.831<br>D_3 0.831<br>D_4 0.85	D_1 0.829<br>D_2 0.831<br>D_3 0.831<br>D_4 0.85	D_1 0.829<br>D_2 0.831<br>D_3 0.831<br>D_4 0.85
**Symmetry imposed**	C1	C1	C1	C1	C1	C1
**Initial particle images (no.)**	1,138,825	1,138,825	D_1 737,138<br>D_2 1,058,026<br>D_3 1,330,232<br>D_4 722,870	D_1 737,138<br>D_2 1,058,026<br>D_3 1,330,232<br>D_4 722,870	D_1 737,138<br>D_2 1,058,026<br>D_3 1,330,232<br>D_4 722,870	D_1 737,138<br>D_2 1,058,026<br>D_3 1,330,232<br>D_4 722,870
**Particle images before merging (no.)**	N/A	N/A	D_1 332,373<br>D_2 280,769<br>D_3 91,929<br>D_4 269,574	D_1 332,373<br>D_2 280,769<br>D_3 91,929<br>D_4 269,574	D_1 332,373<br>D_2 280,769<br>D_3 91,929<br>D_4 269,574	D_1 332,373<br>D_2 280,769<br>D_3 91,929<br>D_4 269,574
**Final particle images (no.)**	206,782	206,782	47,244	33,138	47,244	33,138
**Map resolution (Å)**<br>FSC threshold 0.143	3.0	2.9	3.3	3.3	3.5	3.2
**Map resolution range (Å)**	2.3-8.0	2.3-8.0	3.0-12.4	3.0-10.2	3.1-11.0	3.0-8.0
**Refinement**						
**Initial model used (PDB code)**	7NYD, 2J8B	7NYD,2J8B	7NYD, 2J8B	7NYD, 2J8B	7NYD, 2J8B	7NYD, 2J8B
**Model resolution (Å)**						
FSC threshold 0.5	3.0		3.3	3.2		
**Map sharpening** *B* factor (Å^2)	-60	-60	-50	-50	-50	-50
**Model composition**						
Non-hydrogen atoms	30,943		37,288	40,505		
Protein residues	3910		4704	5113		
Ligands	BMA: 1		BMA: 4	BMA: 0		
Ions	NAG: 6<br>Ca: 2		NAG: 8<br>0	NAG: 10<br>0		
***B* factors (Å^2)**						
**Protein**	86.80		117.93	118.03		
**Ligand**	136.15		187.03	138.05		
**R.m.s. deviations**						
Bond lengths (Å)	0.003		0.001	0.009		
Bond angles (°)	0.739		0.867	0.791		
**Validation**						
MolProbity score	1.81		1.87	1.66		
Clashscore	4.43		8.53	5.49		
Poor rotamers (%)	1.4		0.56	0.31		
**Ramachandran plot**						
Favored (%)	92.24		95.87	94.80		
Allowed (%)	7.76		5.13	5.20		
Disallowed (%)	0.0		0.0	0.0		

Supplementary Table 2. CG parameters of the molecules used for parametrization of the EtNP linker.

Bead name	Bead type	Bond	r_0 (nm)	K_b (kJ/mol)	Angle	θ_0 (°)	K_a (kJ/mol)
T	SN0	T – BB_1	0.3	20000	T – BB_1 – BB_2	128	20.0
BB_1	P5	BB_1 – $SC1_1$	0.33	7500	T – BB_1 – $SC1_1$	108	30.0
$SC1_1$	C1	BB_1 – BB_2	0.39	20000	BB_1 – BB_2 – BB_3	132	30.0
BB_2	P5	BB_2 – $SC1_2$	0.4	5000	$SC1_2$ – BB_2 – BB_3	108	30.0
$SC1_2$	Qa	BB_2 – BB_3	0.35	20000	BB_2 – BB_3 – $SC1_3$	96	80.0
BB_3	P5	BB_3 – $SC1_3$	0.32	5000	BB_2 – BB_3 – L1	105	2.5
$SC1_3$	P5	BB_3 – L1	0.33	10000	$SC1_3$ – BB_3 – L1	90	1.0
L1	GSNda	L1 – L2	0.35	5000	BB_3 – L1 – L2	130	60.0
L2	GQa	L2 – C15	0.365	18000	L1 – L2 – C15	45	5.0
C15	GNa	C15 – C14	0.33	20000	L2 – C15 – C13	180	15.0
C14	GP3	C15 – C13	0.35	20000	L2 – C15 – C14	70	20.0
C13	GSP1	C14 – C13	0.28	40000	C15 – C13 – C10	65	45.0
C12	GP2	C13 – C10	0.36	20000	C14 – C13 – C10	95	100.0
C11	GP3	C12 – C11	0.33	30000			
C10	GSN0	C12 – C10	0.35	30000			
		C11 – C10	0.28	40000			

Dihedral	Type	ϕ_0 (°)	K_d (kJ/mol)
BB_2 – BB_3 – L1 – L2	Proper	220	1.5
BB_3 – L1 – L2 – C15	Proper	–195	4.0
L1 – L2 – C15 – C13	Proper	320	3.5
L2 – C15 – C13 – C10	Proper	30	3.0
BB_1 – BB_2 – BB_3 – L1	Improper	–200	3.0
