# Supplementary material for: Structural basis for membrane attack complex inhibition by CD59
Source: Nat Commun. 2023 Feb 16;14:890. doi: 10.1038/s41467-023-36441-z (PMC9935631; doi:10.1038/s41467-023-36441-z)
Supplement: Supplementary file 3 — Description to Additional Supplementary Information [file 41467_2023_36441_MOESM3_ESM.pdf]

Description of Additional Supplementary Files

Supplementary Movie 1: 3D variability analysis of the focus refined C5b8-CD59 map. A linear movie of volumes visualizing variability across the first principal component is shown. Volumes are coloured according to protein components: CD59 is cyan, C8a is pink, all other proteins in the map are grey

Supplementary Data File Legends:

Supplementary Data File 1: The initial configuration from the atomistic CD59 production run in Gromacs format (first replicate).

Supplementary Data File 2: The final configuration from the atomistic CD59 production run in Gromacs format (first replicate).

Supplementary Data File 3: The initial configuration from the atomistic CD59 production run in Gromacs format (second replicate).

Supplementary Data File 4: The final configuration from the atomistic CD59 production run in Gromacs format (second replicate).

Supplementary Data File 5: The initial configuration from the atomistic CD59 production run in Gromacs format (third replicate).

Supplementary Data File 6: The final configuration from the atomistic CD59 production run in Gromacs format (third replicate).

Supplementary Data File 7: Initial configuration from the coarse-grain CD59-C5b8 production run in Gromacs format (first replicate).

Supplementary Data File 8: Final configuration from the coarse-grain CD59-C5b8 production run in Gromacs (first replicate).

Supplementary Data File 9: Initial configuration from the coarse-grain CD59-C5b8 production run in Gromacs format (second replicate).

Supplementary Data File 10: Final configuration from the coarse-grain CD59-C5b8 production run in Gromacs (second replicate).

Supplementary Data File 11: Initial configuration from the coarse-grain CD59-C5b8 production run in Gromacs format (third replicate).

Supplementary Data File 12: Final configuration from the coarse-grain CD59-C5b8 production run in Gromacs (third replicate).
