# Supplementary material for: Structural basis for membrane attack complex inhibition by CD59
Source: Nat Commun. 2023 Feb 16;14:890. doi: 10.1038/s41467-023-36441-z (PMC9935631; doi:10.1038/s41467-023-36441-z)
Supplement: Supplementary file 17 — Reporting Summary [file 41467_2023_36441_MOESM17_ESM.pdf]

nature research

Corresponding author(s): Doryen Bubeck

Last updated by author(s): Jan 16, 2023

Reporting Summary

Nature Research wishes to improve the reproducibility of the work that we publish. This form provides structure for consistency and transparency in reporting. For further information on Nature Research policies, see our Editorial Policies and the Editorial Policy Checklist.

Statistics

For all statistical analyses, confirm that the following items are present in the figure legend, table legend, main text, or Methods section.

n/a	Confirmed	
☐	☒	The exact sample size (*n*) for each experimental group/condition, given as a discrete number and unit of measurement
☐	☒	A statement on whether measurements were taken from distinct samples or whether the same sample was measured repeatedly
☒	☐	The statistical test(s) used AND whether they are one- or two-sided<br>*Only common tests should be described solely by name; describe more complex techniques in the Methods section.*
☒	☐	A description of all covariates tested
☒	☐	A description of any assumptions or corrections, such as tests of normality and adjustment for multiple comparisons
☐	☒	A full description of the statistical parameters including central tendency (e.g. means) or other basic estimates (e.g. regression coefficient) AND variation (e.g. standard deviation) or associated estimates of uncertainty (e.g. confidence intervals)
☒	☐	For null hypothesis testing, the test statistic (e.g. *F*, *t*, *r*) with confidence intervals, effect sizes, degrees of freedom and *P* value noted<br>*Give P values as exact values whenever suitable.*
☒	☐	For Bayesian analysis, information on the choice of priors and Markov chain Monte Carlo settings
☒	☐	For hierarchical and complex designs, identification of the appropriate level for tests and full reporting of outcomes
☒	☐	Estimates of effect sizes (e.g. Cohen's *d*, Pearson's *r*), indicating how they were calculated

Our web collection on statistics for biologists contains articles on many of the points above.

Software and code

Policy information about availability of computer code

Data collection	CryoEM were collected with EPU software version 1.12.079 (Thermofisher Scientific). Fluorescence images were collected using Leica Application Suite X. Molecular dynamics simulation systems were built using LEaP in Ambertools21, ACPYPE, martinize.py (Python 3.8.8), insane.py (Python 2.7.5), and CHARMM-GUI. Simulations were run using Gromacs 2021.3. We used the following forcefields: Martinti 2.2 with ELNEDYN framework, CHARMM36m, and GLYCAM06h.
Data analysis	CryoEM data was analyzed with published softwares RELION v3.1, cryoSPARC v3.1.1 and v2, CTFFIND4 v4.1, MotionCor2, Gctf v1.06, crYOLO v1.7.6, csparc2star.py (Python 3.6.5), CryoDRGN, ISOLDE v1.1.0, Phenix v1.2, ChimeraX v1.1, AlfphaFold2, and the online portal for SWISSMODEL. Fluorescence images were analyzed using Fiji-2. Molecular dynamics simulations were analyzed using Gromacs 2021.3 and ChimeraX v1.1.

For manuscripts utilizing custom algorithms or software that are central to the research but not yet described in published literature, software must be made available to editors and reviewers. We strongly encourage code deposition in a community repository (e.g. GitHub). See the Nature Research guidelines for submitting code & software for further information.

Data

Policy information about availability of data

All manuscripts must include a data availability statement. This statement should provide the following information, where applicable:

- Accession codes, unique identifiers, or web links for publicly available datasets
- A list of figures that have associated raw data
- A description of any restrictions on data availability

Data supporting the findings of this manuscript are available from the corresponding authors upon reasonable request. A reporting summary for this article is available as a Supplementary Information file. Source data are provided with this paper. Raw data generated in this study underlying Supplementary Fig. 1B and

Supplementary Fig. 8E are included as a Source Data file. Initial and final configurations for MD simulations are included as Supplementary Data Files. The cryoEM maps generated in this study have been deposited in the Electron Microscopy Data Bank under the accession codes EMD-15779 [https://www.ebi.ac.uk/pdbe/entry/emdb/EMD-15779] (C5b8-CD59), EMD-15781 [https://www.ebi.ac.uk/pdbe/entry/emdb/EMD-15781] (C5b92-CD59), and EMD-15780 [https://www.ebi.ac.uk/pdbe/entry/emdb/EMD-15780]
(C5b93-CD59). The structural models generated in this study have been deposited in the Protein Data Bank under the accession codes 8H0F [http://doi.org/10.2210/pdb8h0f/pdb] (C5b8-CD59), 8B0H [http://doi.org/10.2210/pdb8b0h/pdb] (C5b92-CD59), and 8B0G [http://doi.org/10.2210/pdb8b0g/pdb] (C5b93-CD59). Structural models used to initiate model building were accessed from the Protein Data Bank under the accession codes 7NYD [http://doi.org/10.2210/pdb7nyd/pdb], 2J8B [http://doi.org/10.2210/pdb2j8b/pdb] and 6H03 http://doi.org/10.2210/pdb6h03/pdb]. Structural models used in data analysis were accessed from the Protein Data Bank under the accession codes 5IMT [http://doi.org/10.2210/pdb5imt/pdb], 7NYD [http://doi.org/10.2210/pdb7nyd/pdb], 6H03 [http://doi.org/10.2210/pdb6h03/pdb], and from the AlphaFold protein structure database entry O5518 [https://alphafold.ebi.ac.uk/entry/O55186]. Structural models used to generate Fig.1 were accessed from the Protein Data Bank under the accession codes 6H03 [http://doi.org/10.2210/pdb6h03/pdb], 3T5O [http://doi.org/10.2210/pdb3t5o/pdb], 3OJY [http://doi.org/10.2210/pdb3ojy/pdb], 6CXO [http://doi.org/10.2210/pdb6cxo/pdb], and from the AlphaFold protein structure database entry P10643 [https://alphafold.ebi.ac.uk/entry/ P10643].

Field-specific reporting

Please select the one below that is the best fit for your research. If you are not sure, read the appropriate sections before making your selection.

☒ Life sciences ☐ Behavioural & social sciences ☐ Ecological, evolutionary & environmental sciences

For a reference copy of the document with all sections, see nature.com/documents/nr-reporting-summary-flat.pdf

Life sciences study design

All studies must disclose on these points even when the disclosure is negative.

Sample size	For structural studies of C5b9-CD59 complexes, 974,249 particles were picked from 52,838 electron micrograph movies. For structural studies of C5b8-CD59 complex, 1,138,825 particles were picked from 12,805 micrograph movies. For fluorescence microscopy experiments, 10 randomly located regions were collected for each condition and 10-20 individual cells were analyzed. No sample size calculations were performed for the cryoEM data or the fluorescence microscopy data. The cryoEM data collected is sufficient for the resolution of the reported maps. No statistical analysis is performed for the location of MAC in the fluorescence images; therefore the sample size chosen is sufficient.
Data exclusions	For structural studies, electron micrograph movies with substantial drift and crystalline ice were excluded. This is a pre-established standard in the cryoEM community. C5b8-Cd59 and C5b9-CD59 picked particles were excluded based on 2D and 3D classification. Excluded particles were those which belonged to classes that lacked high resolution structural features; this is a pre-established standard in the cryoEM community. For C5b9-CD59, particles from uninhibited classes were removed to improve the homogeneity of the reconstruction. Classes that were consistent with either 2 3 or 4 copies of C9 were taken forward for further analysis.
Replication	MD simulations were performed for three independent replicates. Cholesterol depletion assay was performed for three technical replicates. All attempts at replication were successful.
Randomization	Not relevant to this study, since samples were not allocated into experimental groups.
Blinding	Not relevant to this study, since there were no group allocations in this study.

Reporting for specific materials, systems and methods

We require information from authors about some types of materials, experimental systems and methods used in many studies. Here, indicate whether each material, system or method listed is relevant to your study. If you are not sure if a list item applies to your research, read the appropriate section before selecting a response.

Materials & experimental systems

n/a	Involved in the study	
☐	☒	Antibodies
☐	☒	Eukaryotic cell lines
☒	☐	Palaeontology and archaeology
☒	☐	Animals and other organisms
☒	☐	Human research participants
☒	☐	Clinical data
☒	☐	Dual use research of concern

Methods

n/a	Involved in the study	
☒	☐	ChIP-seq
☒	☐	Flow cytometry
☒	☐	MRI-based neuroimaging

Antibodies

Antibodies used	rabbit anti-CHO polyclonal IgG (catalogue number: 27803-1-AP Proteintech) was used to activate complement
Validation	Validated by the manufacturer (https://www.ptglab.com/products/CHO-cells-Antibody-27803-1-AP.htm)

Eukaryotic cell lines

Policy information about cell lines

Cell line source(s)	CHO-K1 cells were a gift from A. Kusumi
Authentication	The cells were not authenticated.
Mycoplasma contamination	The cells were tested for Mycoplasma and found to be negative.
Commonly misidentified lines (See ICLAC register)	*Name any commonly misidentified cell lines used in the study and provide a rationale for their use.*
